# Ankrd2 in Mechanotransduction and Oxidative Stress Response in Skeletal Muscle: New Cues for the Pathogenesis of Muscular Laminopathies

**DOI:** 10.1155/2019/7318796

**Published:** 2019-07-24

**Authors:** Vittoria Cenni, Snezana Kojic, Cristina Capanni, Georgine Faulkner, Giovanna Lattanzi

**Affiliations:** ^1^CNR National Research Council of Italy, Institute of Molecular Genetics, Unit of Bologna, 40126 Bologna, Italy; ^2^IRCCS Istituto Ortopedico Rizzoli, 40126 Bologna, Italy; ^3^Institute of Molecular Genetics and Genetic Engineering University of Belgrade, 11010 Belgrade, Serbia; ^4^Department of Biology, University of Padua, 35121 Padua, Italy

## Abstract

Ankrd2 (ankyrin repeats containing domain 2) or Arpp (ankyrin repeat, PEST sequence, and proline-rich region) is a member of the muscle ankyrin repeat protein family. Ankrd2 is mostly expressed in skeletal muscle, where it plays an intriguing role in the transcriptional response to stress induced by mechanical stimulation as well as by cellular reactive oxygen species. Our studies in myoblasts from Emery-Dreifuss muscular dystrophy 2, a *LMNA*-linked disease affecting skeletal and cardiac muscles, demonstrated that Ankrd2 is a lamin A-binding protein and that mutated lamins found in Emery-Dreifuss muscular dystrophy change the dynamics of Ankrd2 nuclear import, thus affecting oxidative stress response. In this review, besides describing the latest advances related to Ankrd2 studies, including novel discoveries on Ankrd2 isoform-specific functions, we report the main findings on the relationship of Ankrd2 with A-type lamins and discuss known and potential mechanisms involving defective Ankrd2-lamin A interplay in the pathogenesis of muscular laminopathies.

## 1. Introduction

Ankrd2 (ankyrin repeats containing domain 2) or Arpp (ankyrin repeat, PEST sequence, and proline-rich region) is a member of the MARP (muscle ankyrin repeat protein) family of proteins that also includes Ankrd1, or Carp (Cardiac Ankyrin Repeat Protein), and Ankrd23, or DARP (Diabetes-related Ankyrin Repeat Protein) [1]. As we will describe in detail in this review, Ankrd2 is principally expressed in skeletal muscle, where it plays an intriguing role in the transcriptional response to stress induced by mechanical stimulation, as well as by cellular ROS (reactive oxygen species). In particular, although the Ankrd2 knockout mouse model has revealed that Ankrd2 is not necessary for life nor the direct cause of any muscular disorder, multiple evidence obtained from muscular cell lines or primary cultures from patients affected by muscular diseases suggest that an anomalous expression level of Ankrd2, as well as defective nuclear recruitment of the protein, might contribute to a muscular phenotype. Very recently, we found that Ankrd2 is a novel interactor of A-type lamins, which are produced by the *LMNA* gene and are the major constituents of the nuclear lamina. Mutations of *LMNA* gene are the cause of a substantial number of pathologies, some of which affecting skeletal and cardiac muscles. Moreover, altered expression of Ankrd2 was detected in myopathies [2], cardiomyopathies [3], and some tumors [4]. In this review, apart from describing the latest advances related to Ankrd2, including an ever-growing interest for Ankrd2 isoform-specific functions, we will report the main findings on Ankrd2 and its relation to lamins and discuss known and potential mechanisms, through which defective Ankrd2-lamin A interplay might have a role in the pathogenesis of *LMNA*-related muscular disorders.

## 2. Ankrd2 in Striated Muscles

Ankrd2, together with other proteins of the MARP family, is considered as STRaND (shuttling transcriptional regulators and non-DNA-binding protein), having signature features: shuttling from the cytoplasm to the nucleus, absence of DNA binding, and regulation of transcription via interaction with transcription factors [5]. Recently, data from Wette et al., obtained on single muscle fibers, have shown that Ankrd2 is mostly found (>70% of total Ankrd2) in the cytosol [6]. The remaining amount is distributed between intracellular structures, including the sarcomere and the nucleus, with proportions that reflect the physiology, such as the type (slow/fast) or the condition (forming/mature/regenerating/injured), of the fiber considered. In healthy striated muscle, Ankrd2 is found at the sarcomeric I-band [7, 8] where it contributes to the formation and function of the I-band mechanosensory complex, probably via interaction with calpain 3 and the elastic portion of titin (N2A region) [7, 9]. As extensively described in the next sections, Ankrd2 also interacts with nuclear proteins with both structural and enzymatic roles [1] and is accumulated in nuclei of injured muscle [10]. An overview of the main structural and functional features of Ankrd2, including protein domains, posttranslational modifications, expression profile, proposed functions, interacting proteins, and pathways affected by *ANKRD2* silencing in human myoblasts, is given in Table 1. In the nucleus, Ankrd2 participates in the regulation of genes involved in the cell cycle, myogenesis, and inflammatory response [11–15]. Of note, in mature muscle cells, the evidence reporting that the majority of Ankrd2 is freely diffusible in the cytosol [6] has for the first time pointed to the open question if Ankrd2, besides being involved in sarcomeric activity, might also participate in cellular functions in other subcellular districts, thus suggesting a role as a downstream signal transducer in mechanosignaling pathways. It is important to note that despite accumulated findings from cell culture experiments, direct evidence of Ankrd2 function in mature healthy and diseased striated muscles is still missing.

### 2.1. Ankrd2 Expression in Skeletal Muscle

Ankrd2 was reported for the first time in a study performed in 2000 by Kemp and colleagues as a protein upregulated in murine muscle exposed to prolonged passive stretch (7 days) [16]. Preferential expression in skeletal muscle, more specifically in slow (type I) muscle fibers, and responsiveness to mechanical stress are signature features of this protein. Recently, Ankrd2 was confirmed to be the most abundant MARP protein in human skeletal muscle, being ~30-fold more expressed than DARP and ~150-fold more than Ankrd1 [6]. In this study, performed on a group of four subjects, the absolute amount of Ankrd2 was subject-specific, ranging from 1.6 to 4.3 *μ*mol/kg net weight, and proportional to the content of myosin heavy chain I (MHCI) protein, which is a marker of slow (type I) muscle fibers [6]. Almost double the amount of Ankrd2 was measured in slow (type I) fibers compared to that of fast (type II) fibers microdissected from the *vastus lateralis* (presenting approximately 32% of slow muscle fibers [17]), corroborating Ankrd2 preferential expression in slow fibers [6].

Although the existence of Ankrd2 isoforms was reported in 2003 by Miller et al. [7], their specific expression and localization in human skeletal and cardiac muscles, as well as in primary human myoblasts and myotubes, have been recently determined by studies in which their discrimination was enabled by specific antibodies [6, 18].

**Figure 1:**
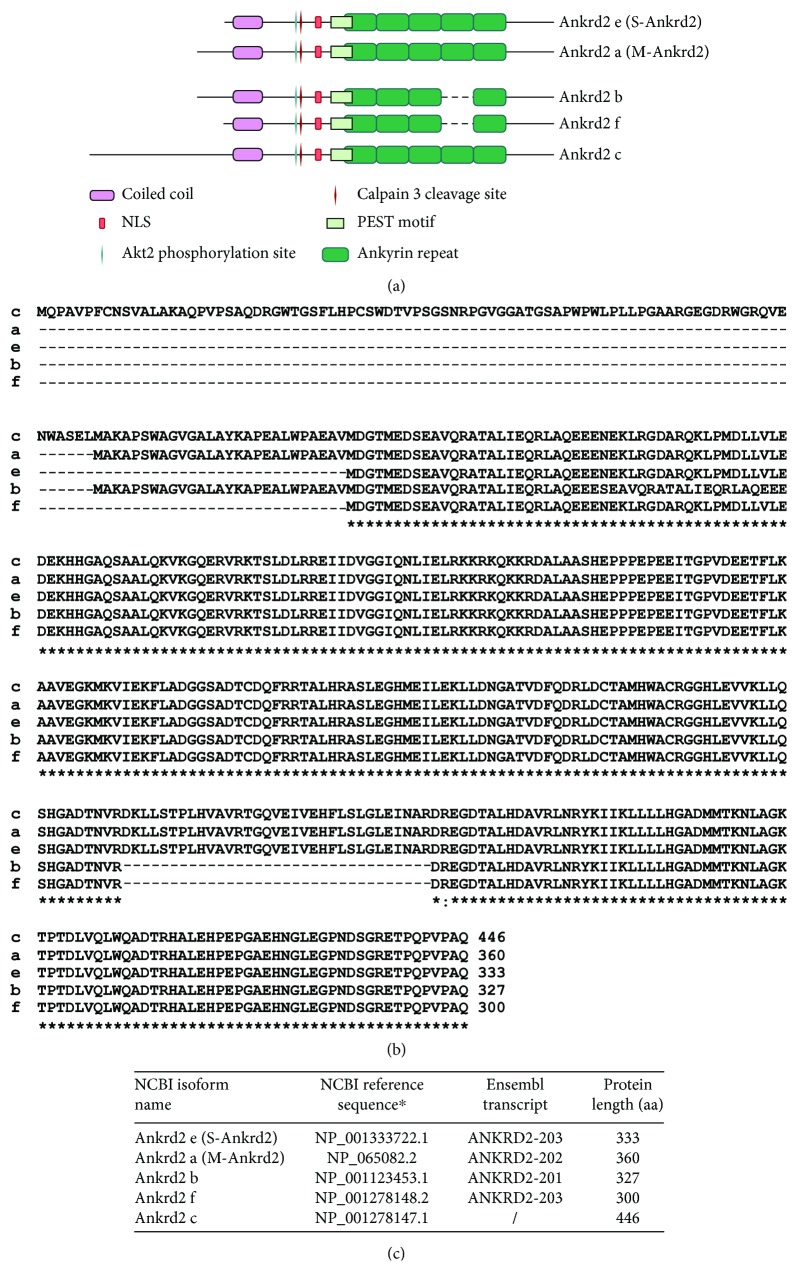
Overview of Ankrd2 isoforms. (a) Schematic presentation of the Ankrd2 domain structure. (b) Alignment of protein sequences of Ankrd2 isoforms. Numbers correspond to amino acids. (c) Entries for five Ankrd2 isoforms reported in NCBI and Ensembl databases. ^∗^NCBI entries were updated in 2018.

The most important Ankrd2 isoforms, namely, S-Ankrd2 (333 aa) and M-Ankrd2 (360 aa), are identical, except for 27 aa extension at the N-terminus of M-Ankrd2. According to qPCR results, the expression of Ankrd2 isoforms is regulated at the transcriptional level [18]. S-Ankrd2 is expressed in both heart and skeletal muscles [18] where it is the predominant form [6, 18]. The well-known chessboard pattern of Ankrd2 expression observed in transversal sections of skeletal muscle may be attributed almost exclusively to S-Ankrd2 [6, 18], which has been therefore proposed as the canonical Ankrd2 isoform. Although still a matter of debate, in mature muscle fibers, the small amount of M-Ankrd2 has been identified in nuclei [18]. In human myoblasts, S-Ankrd2 is expressed mainly in nuclei at a low level, while in myotubes, it is upregulated and localized in the cytoplasm. M-Ankrd2 is also upregulated upon differentiation of myoblasts to myotubes; however, it maintains its nuclear localization. Striking differences in expression and localization of Ankrd2 isoforms may mirror their different functions which however need to be further explored [18]. For example, M-Ankrd2 might have a more regulatory role, being predominantly nuclear, while sarcomeric and cytosolic S-Ankrd2 may have a structural and downstream signaling role, respectively. Domain organization, alignment, and updated database entries for all five Ankrd2 isoforms are shown in Figures 1(a)–1(c), respectively.

### 2.2. Ankrd2 Expression in Cardiac Muscle

Ankrd2 is expressed in the human heart, including ventricles, the interventricular septum, and the apex of the heart [4, 19], at a lower level compared to skeletal muscle. Ankrd2 expression in the heart is potentially regulated by cardiac-specific transcription factors Nkx2.5, Hand2, and Ankrd1 as demonstrated by their interaction with the *ANKRD2* promoter or by dual luciferase assay [20, 21]. Transcriptome profiling confirmed a lower level of Ankrd2 in the ventricles of human adult heart, compared to Ankrd1 [22]. Both S- and M-Ankrd2 isoforms are expressed in cardiac muscle; they have sarcomeric localization and are almost absent from nuclei [18]. The latter finding and the known interaction between Ankrd2 and the gap junction protein ZO-1 [20], enriched in ICDs, suggest a role for Ankrd2 in intercellular communication, which merits further investigation. In neonatal rat cardiomyocytes (NRCM), both sarcomeric and nuclear localizations of Ankrd2 were investigated during cellular differentiation and not shown to be affected by the maturation stage [22]. Moreover, doxorubicin had no effect on Ankrd2 expression, as opposed to Ankrd1, suggesting that Ankrd2 is not involved in cellular response to this cardiotoxic antineoplastic drug.

It is interesting that, in addition to mild phenotype in skeletal muscle, morphology, basal cardiac function, and cardiac development were not impaired in triple knockout mice for three members of the MARP family (Ankrd1, Ankrd2, and DARP) up to 17 months of age [23]. Hypertrophic response to acute pressure overload induced by transverse aortic constriction was also unaffected. It is possible that Ankrd2 is involved in the response to other types of cardiac stress, still to be determined. On the other hand, as demonstrated for Ankrd1, overexpression of Ankrd2 may have more striking consequences. Overexpressed mouse Ankrd1 is indeed involved in inhibition of cardiac hypertrophy and development of fibrosis in response to pressure overload- and isoproterenol-induced cardiac hypertrophy [24].

Apart from altered expression in skeletal muscle disorders, Ankrd2 was found upregulated in dilated cardiomyopathy patients [3]. Together with several titin ligands associated with sarcomeric mechanosensory complexes, the expression pattern of Ankrd2 was determined in endomyocardial biopsies of patients with advanced idiopathic dilated cardiomyopathy (IDCM) characterized by extensive remodeling of the left ventricle [25]. Expression of Ankrd2, as well as Ankrd1 and TRIM63, was induced and in correlation with the disease stage. Ankrd2 expression was significantly higher in patients with severe symptoms, compared to patients with moderate symptoms. Although this finding has no clinical significance, experimental evidence strongly supports the involvement of Ankrd2 in pathological cardiac remodeling [25].

### 2.3. Ankrd2 and Mechanotransduction in Skeletal Muscle

Mechanotransduction is the ability of cells to sense external mechanical forces, create biochemical intracellular signals, and trigger the expression of specific genes that drive the adaptation to mechanical changes. An incorrect mechanotransduction may affect muscle differentiation, myofiber turnover, and stem cell renewal [26].

**Figure 2:**
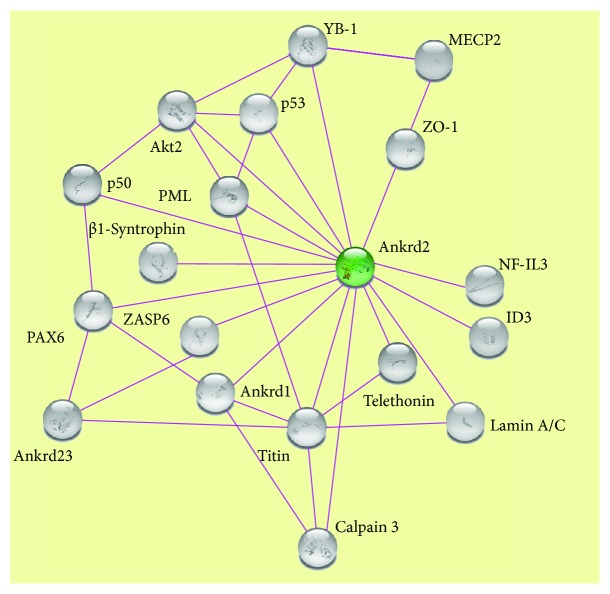
Interaction network for Ankrd2. Interactors listed in Table 1 were loaded into the STRING database: https://string-db.org/. The majority of Ankrd2 interactors, including titin, telethonin, Ankrd1, Ankrd23, ZASP6, and calpain 3, are involved in mechanosignaling. Other Ankrd2 interacting proteins are involved in DNA binding and regulation of transcription and include p53, MECP2, ID3, YB-1, PAX6, PML, and NF-IL3. A-type lamins, including lamin A, its precursor prelamin A, and lamin C, are Ankrd2 interactors and are involved in multiple cellular functions, including modulation of the nuclear stiffness in response to mechanical and oxidative stress and of epigenetic factor activity. Ankrd2 also interacts with protein kinases, as Akt2, and with proteins with PDZ or SH3 domains able to mediate protein-protein interactions (including ZO-1 and *β*1-syntrophin, respectively). Note that the majority of Ankrd2 interactors were identified from in vitro studies or in nonmuscular cellular systems (see text and Table 1 for references).

Ankrd2 plays an important role in sensing mechanical changes in skeletal myofibers. It is found at the elastic I-band segment of the sarcomere, where a well-defined signalosome, composed by several structural and regulatory proteins, resides [10]. In vitro studies suggest that Ankrd2 might be assembled on the N2A spring element of the giant protein titin [7]. Titin is the largest of mammalian proteins, spanning over half of a sarcomere, with its N-terminus located in the Z-disc and its C-terminus located in the M-line. In striated muscle, one of titin's classical roles is to provide elasticity to sarcomeres [27]. Titin is characterized by multiple domains that interact with several sarcomeric proteins and also with enzymes regulating its own properties and resulting in the modulation of its stiffness. In humans, mutations or partial deletion of titin might cause muscular dystrophies, such as Tibial Muscular Dystrophy (TMD) [28] or limb-girdle muscular dystrophy 2J (LGMD-2J) [29]. In vitro evidence demonstrates that besides titin [7], Ankrd2 might also interact with calpain 3 [9] and Z-disc proteins telethonin/TCAP [1] and ZASP6 [30]. The interaction network of Ankrd2 is presented in Figure 2. In particular, calpain 3, a Ca^2+^-dependent protease, coordinates signal transduction from other sarcomeric regions, including Z-disc and M-line where other signaling complexes are found. Interestingly, in the HEK293 cell line, calpain 3 binds and cuts murine Ankrd2 at the Arg104 of its PEST sequence [31] suggesting that calpain 3-mediated proteolysis of Ankrd2 might have a role in Ankrd2 sarcomeric sequestration. However, since these findings have been obtained on recombinant proteins or by using nonmuscular cellular models [9, 31] and since in skeletal muscle, the access of calpain 3 to substrates is restricted, being the majority of the enzyme sequestered by titin [32], results on Ankrd2 proteolysis by calpain 3 in muscular cells still need a proper validation.

Insufficient Ankrd2 recruitment at the I-band has been observed in exercised mice affected by a progressive muscular dystrophy due to an inactivation of calpain 3 (CAPN3-KI mice) [33]. Although this study does not provide sufficient proofs of Ankrd2 proteolysis by calpain 3, for example, loss of Ankrd2 or cleavage to a lower band, the altered Ankrd2 localization in CAPN3-KI muscle suggests that calpain 3 protease activity might be involved (directly or indirectly) in Ankrd2 recruitment at the sarcomere. The importance of a functional signalosome composed of Ankrd2 (as well as other MARPs), calpain 3, and the N2A region of titin is also observed in mdm (muscular dystrophy with myositis) mice, a model for human TMD, caused by the deletion of a large portion of the N2A region, bound by calpain 3. Homozygous mdm mice show a severe and progressive muscular degeneration. Interestingly, Ankrd2 and other MARPs are more abundantly expressed in the skeletal muscles of mdm when compared to wild-type mice [34]. In vitro, Ankrd2 also interacts with Z-disc proteins, including telethonin/TCAP [1] and ZASP6 [30]. Z-disc is involved in conveying mechanical signals and acts as a signaling hub for communication with the nucleus. On the other hand, telethonin links titin to the Z-disc [35]. Mutation of telethonin encoding gene (*TCAP*) may cause intense biomechanical stress, maladaptation, apoptosis, and global heart failure, as well as LGMD-2G [36]. ZASP has several isoforms containing PDZ and LIM domains [30]. Interestingly, in vitro studies have demonstrated that point mutations in the *ZASP6* gene causative for a muscular disease called ZASPopathy prevented ZASP6 interaction with Ankrd2, opening the question of Ankrd2 involvement in the pathophysiology of this myofibrilar myopathy [30]. Myopalladin is a Z-disc protein involved in sarcomere assembly and regulation of gene expression and similarly to Ankrd2 is involved in bringing sarcomeric signals to the nucleus of muscle cells [37]. It is intriguing that, despite interacting with both Ankrd1 and Ankrd23 [7], myopalladin was not shown to bind Ankrd2, neither in vivo nor in vitro. Sarcomeric signalosomes have to be considered as highly dynamic complexes. Their protein components are subjected to a multitude of posttranslational modifications (PTMs) which might affect peculiar properties including their availability and stability, accessibility to interactors, turnover, and intracellular localization. This is the case, for example, of the binding between members of Z-disc FATZ (calsarcin/myozenin), myotilin, and Enigma families, which are modulated by phosphorylation [38]. As a matter of fact, in addition to structural proteins, sarcomeric signalosomes also contain protein-modifying enzymes, including protein kinases (PKC alpha, CaMKII *δ*, Erk1/2, and PKA [39]), E3 ubiquitin ligases (Mdm2 [40], TRIM63/MURF1 [41]), and autophagic protein carriers (p62/SQSTM1 [42]). Therefore, genetic mutations of both structural and enzymatic proteins of the sarcomere, causing a gain or a loss of function, may result in an impaired sensing of the mechanical stress and might be responsible for an inadequate transcriptional response, with harmful consequences for cell physiology.

Accordingly, also defects in Ankrd2 PTMs, including phosphorylation or proteolysis, by altering Ankrd2 binding to specific interactors or intracellular localization, might elicit pathogenetic effects.

Combining the aforementioned information together, we can hypothesize a scenario in which in muscle cells, Ankrd2 might cover multiple roles: a sensor one at the I-band of the sarcomere, where an Ankrd2 fraction might sense the stress, and a transducer one distributed between the cytoplasm, where the more abundant pool of Ankrd2 resides [7], and the nucleus of muscle cells [37] where a fraction of Ankrd2 is detected following stress condition. By mechanisms involving phosphorylation or other PTMs, Ankrd2 might dynamically translocate from the sarcomere, to the cytoplasm and to the nucleus and *vice versa*, participating in the integration of signals derived from mechanosensors or activated by oxidative stress, resulting in the modulation of transcriptional response to stress.

However, since the majority of the interactions between Ankrd2 and sarcomeric and nonsarcomeric proteins have been identified using recombinant proteins and in nonmuscular cellular systems, until their validation on skeletal muscle, the functional relevance of Ankrd2 association with these proteins remains to be demonstrated, and the role and the involvement of Ankrd2 in the physiology of muscle cells might at the moment be only speculated.

## 3. Ankrd2 in the Interplay between Oxidative and Mechanical Stresses

Physical exercise, including moderate and intense muscular activity, results in an increase of ROS. During or after aerobic exercise, as well as anaerobic exercise, the general increase of substrate utilization combined with the increase of oxygen uptake results in the activation of specific metabolic pathways, triggering the generation of ROS [43]. Although the exact redox mechanisms underlying the formation of exercise-induced ROS remain elusive to date, mitochondria, glutathione peroxidase, superoxide dismutase, catalase, NADPH oxidases, and xanthine oxidases have all been identified as potential contributors to ROS production. ROS themselves play a controversial role, since at high concentration they are detrimental for muscle biology [44]. However, low doses of ROS are fundamental to drive exercise adaptations, triggering molecular pathways that culminate with angiogenesis, mitochondria biogenesis, and muscle hypertrophy [43]. We hypothesize that upregulation of Ankrd2 in muscle exposed to mechanical stress is at least partly regulated by altered levels of ROS, as a consequence of increased muscle damage and regeneration [45].

### 3.1. Ankrd2 Upregulation in Response to Mechanical and Oxidative Stresses

#### 3.1.1. Mechanical Stress

Although considered as a multitasking protein, the most striking signature of Ankrd2 is its stress-responsive function. Ankrd2 involvement in the physiology of stressed healthy and diseased muscles has been extensively studied. Ankrd2 expression increases in stress conditions caused by the application of mechanical forces. This was reported for the first time in murine *tibialis anterior* muscles exposed to passive stretch [16]. Subsequent studies further suggested that the increase of Ankrd2 upon passive stretch could be involved in the phenotypic adaptation of fast to slow fibers, which is generally related to long-endurance activities [46]. Increase of Ankrd2 expression in both murine and human muscles has also been reported following jumping exercise [47, 48] and eccentric contraction [49, 50] as well as denervation which reproduces muscle morphogenesis [8]. Of note, Ankrd2 expression in murine muscle is not stimulated by isometric contraction [50], while it is downregulated in inactive muscles [51, 52]. Increased expression of Ankrd2 in skeletal muscle in response to mechanical stress seems to be evolutionarily conserved. Recently, we have demonstrated mild upregulation of zebrafish Ankrd2 in skeletal muscle subjected to endurance exercise training by forced swimming [53].

Ankrd2 increase upon stress stimuli probably strengthens its structural and regulatory functions within the sarcomere. Accordingly, upregulation of Ankrd2 by ROS might contribute to initial events in the adaptative remodeling of exposed fibers, possibly by promoting sarcomerogenesis, the switch from fast to slow fibers [46], and the increase of the mechanotransducing activity.

#### 3.1.2. Oxidative Stress

During oxidative stress caused by exposure of myoblasts to H_2_O_2_, Ankrd2 expression is slightly upregulated [15]. From a molecular point of view, Ankrd2 gene activation by ROS might be regulated by the well-known ROS-dependent activation of the transcription factor NF-*κ*B [54]. As a matter of fact, although direct demonstration is missing, it is possible that upon oxidative stress, ROS-dependent increase of Ankrd2 expression occurs through two putative NF-*κ*B-binding sites within the Ankrd2 gene promoter [12, 55]. However, the contribution of muscle-specific transcription factors and the possibility that ROS controls the Ankrd2 level by affecting its protein stability cannot be ruled out. Ankrd2 upregulation in some human dilated cardiomyopathies [3] and congenital myopathies [2] might be also related to an altered response to mechanical strain, as well as to altered levels of ROS reported in these disorders, as a consequence of increased muscle damage and regeneration [45].

### 3.2. Nuclear Translocation of Ankrd2 under Stress Conditions

Apart from increasing the Ankrd2 expression level, oxidative stress also induces Ankrd2 to partly relocate to the nucleus of muscle cells. For example, in differentiated C2C12 muscle cells, Ankrd2 is shuttled in the nucleus upon cellular exposure to ROS [15], following phosphorylation of Ankrd2 at Serine 99 [15].

In mature muscle injured by cardiotoxin injection, Ankrd2 translocates to the nuclei of myofibers located adjacent to severely damaged myofibers [10]. Similarly, Ankrd2 was shown to translocate to the nucleus also in healthy and CAPN3-KI muscles from mice subjected to exercise [33]. Opposite to findings reported for C2C12 muscle cells exposed to ROS, mechanisms ruling Ankrd2 nuclear localization in mature muscle cells remain hitherto to be determined. Further studies are needed to assess if Ankrd2 localization occurs in a fashion similar to that reported for ROS-treated C2C12 [15], involving Ankrd2 PTMs, or by the expression and/or activation of a specific nuclear Ankrd2 form, still to be determined.

Importantly, both nuclear translocation and increased expression of Ankrd2 induced by ROS or myogenic differentiation are related to retardation of myogenic differentiation, allowing cells to properly overcome stress before completing the differentiation program [11, 15]. In human myoblasts, this function is executed via its interaction with DNA-binding proteins, including transcription factors, such as the Y-box transcription factor 1 (YB1), the promyelocytic leukemia protein (PML), and p53 [1], whereas in primary myoblasts from mdm mice, Ankrd2 was shown to physically interact with the inhibitor of DNA-binding protein 3, ID3 [13]. Moreover, upon muscle stress stimulation, Ankrd2 was also shown to colocalize with acetylated histones, which generally mark active chromatin, in the nucleus of myocytes [10].

Nuclear Ankrd2 overexpression induced by ROS has a different outcome in regard to the phase of the cell cycle [11]. In cycling myoblasts, Ankrd2 overexpression induces apoptosis, and this effect is probably mediated by Ankrd2 and p53-dependent upregulation of p21 [1, 11]. On the other hand, Ankrd2 overexpression impairs myogenic differentiation through downregulation of MyoD and myogenin as well as their target Myh1, a terminal differentiation marker [11]. Supporting the altered myogenic program, Ankrd2 overexpressing myotubes also express Myf-5, which is normally expressed in cycling myoblasts [11]. An intriguing, albeit unexplored evidence of the transcriptional role of Ankrd2 came from the studies of triple knockout mice lacking all three MARPs [56]. They expressed a longer titin isoform, hinting at an unexpected link between titin gene expression and MARPs [56].

Studies from our laboratories have demonstrated that Ankrd2 also modulates the activity of NF-*κ*B, through direct interaction with the NF-*κ*B repressor p50 [12]. NF-*κ*B is a transcription factor composed of homo- and heterodimers, affecting the expression of genes related to inflammatory and cell survival responses [57]. The interaction between Ankrd2 and p50 abrogates NF-*κ*B activation, resulting in a negative regulation of its downstream genes. Moreover, p50/p50 homodimer negatively regulates the expression of Ankrd2 by binding its promoter, in a classic feedback inhibition manner [12]. In this condition, we have shown a significant reduction of proinflammatory cytokines, including IL-1*β* and IL-6 [12] that, at high concentrations, may cause proteolysis in muscle and participate in muscle wasting [45, 58].

## 4. Lamin A/C and Muscular Laminopathies

### 4.1. Role of Lamin A/C in Muscular Cellular Physiology

A-type lamins, including lamin A and lamin C, are the main products of the *LMNA* gene and are the major constituents of the nuclear lamina, a filamentous meshwork underneath the nuclear envelope that can be found in all vertebrate cells [59]. Lamin A is transcribed and translated as a precursor protein, prelamin A, which undergoes complex posttranslational processing [59]. Final cleavage by the protease ZMPSTE24 (zinc metallopeptidase STE24) eliminates the last 15 amino acids of prelamin A, releasing a short peptide and mature lamin A [60]. Notably, prelamin A and its processing pathway have been implicated in both physiological and pathogenic mechanisms [59].

Prelamin A increase occurs during the first steps of myoblast differentiation [61], and prelamin A-mediated recruitment of the nuclear envelope proteins SUN1, SUN2 [62], and Samp1 [63] is required for proper myonuclear positioning during myogenic differentiation. In cultured fibroblasts, prelamin A modulation during stress response is a physiological mechanism related to import of DNA repair factors [64] or activation of chromatin remodeling enzymes [65]. However, anomalous prelamin A accumulation, due to specific mutations in the *LMNA* or *ZMPSTE24* genes [59], causes toxicity leading to cellular senescence [66] and represents the molecular basis of progeroid laminopathies [59].

Via interaction with structural and enzymatic molecules, A-type lamins enable several cellular functions, including mechanotransduction [67], shuttling of proteins from the cytoplasm to the nucleus [68], and epigenetic modification of chromatin, affecting the expression of proteins involved in the cell cycle and/or differentiation [69]. The pleiotropic involvement of A-type lamins in all these cellular events explains why mutations in the *LMNA* gene are the cause of a wide number of disorders, collectively known as “laminopathies,” which may affect one or more specific tissues, including skeletal and cardiac muscles, tendons, adipose tissue, and peripheral neurons, or be systemic causing accelerated ageing [70].

One of the most important cellular activities affected by *LMNA* mutations is mechanotransduction [71], a function particularly relevant in striated muscle [26]. From a molecular point of view, mechanical changes propagate from the cytoskeleton to the nuclear membrane and chromatin through direct interaction between A-type lamins and the LINC (linker of nucleoskeleton and cytoskeleton) complex [72], which is formed by SUN1 and SUN2 at the inner nuclear membrane and nesprins, which also span the outer nuclear membrane. Nesprins interact with cytoskeletal actin and, by binding to plectin, with microtubules and intermediate filaments [73]. However, nesprins also interact with lamin A/C [74]. Mutation of each of the components of the LINC complex negatively affects the functions of this intricate mechanism, resulting in an interruption or an alteration of the modulation of mechanoresponse and mechanotransduction of muscle cells [75, 76]. Not surprisingly, LINC protein mutations cause muscular dystrophies including Emery-Dreifuss muscular dystrophy (EDMD) [77–80].

### 4.2. Muscular Laminopathies

Mutations in *LMNA* gene cause a variety of muscular diseases including EDMD, limb-girdle muscular dystrophy 1B (LGMD1B), dilated cardiomyopathy with conduction defects (DCM1A), and *LMNA*-related congenital muscular dystrophy (L-CMD) [60, 81, 82]. Moreover, muscle functionality may be impaired in cases of progeroid laminopathies or lipodystrophies [59, 83]. The inheritance pattern of *LMNA*-related muscle diseases is autosomal dominant and very rarely autosomal recessive. All these disorders are characterized by joint contractures, muscle weakness and wasting, and dilated cardiomyopathy. The L-CMD onset is at birth, whereas EDMD2 and EDMD3 (respectively, with dominant and recessive inheritance) have an onset in childhood or young adulthood, as most cases of LGMD1B. DCM1A, which is usually diagnosed in the second decade, is often associated with conduction disorders and cardiac arrhythmias with life-threatening outcome requiring defibrillator implantation and, in severe cases, heart transplantation [81]. Extracardiac features including skeletal muscle weakness and contractures may occur later in DCM1A, causing a phenotype overlapping with EDMD2 [84].

### 4.3. Pathogenetic Mechanisms in Muscular Laminopathies

*LMNA* gene mutations negatively modulate mechanoresponse and mechanotransduction in muscle cells essentially by two different ways. The first mechanism is related to the structural role played by A-type lamins in the assembly of a molecular platform able to connect and coordinate multiple mechanical and chemical stimuli. The second modality implies alteration of lamin A/C epigenetic activity that results in an abnormal regulation of responsive genes [65]. Cells lacking A-type lamins or expressing myopathic lamin A mutants show reduced nuclear stiffness and altered nuclear morphology and are unable to transmit forces to the nucleus [67]. In addition, the localization of various components of the LINC complex is severely disturbed in those cells [67]. For instance, in EDMD2 myotubes, the mislocalization of SUN1 and SUN2 due to reduced prelamin A interaction was responsible for an altered positioning of myonuclei [62]. The pathogenetic mechanism also involved mislocalization of the nuclear envelope protein Samp1 and defective interplay with the centrosomal protein pericentrin [63]. All these findings highlight the prominent role of A-type lamins in nucleocytoskeleton crosstalk. Importantly, it has been demonstrated that upon mechanical stress, the Ig fold domain of lamin A is able to partially unfold, leading to stretching of the entire molecule [85]. Interestingly, when the extracellular matrix (ECM) increases its stiffness, lamin A undergoes dephosphorylation at Serine 22, a condition enabling increased nuclear stiffness [86]. It is likely that phosphorylation of other residues of lamin A/C contributes to modulation of nuclear stiffness in response to ECM stimuli [87–89]. We found that the pathogenicity of an EDMD2-causative lamin A/C mutation, Arg401Cys, is related to impaired phosphorylation of Serine 404 by Akt1 [90]. It is conceivable that, since phosphorylation of Serine 404 is required for prelamin A and lamin A turnover, impaired phosphorylation might alter nuclear lamina composition and stiffness in response to mechanical stimuli [90, 91].

Altered chromatin organization is considered as a main pathogenic condition in muscular laminopathies [60]. Both high-order chromatin structure [92] and promoter-specific epigenetic defects lead to altered gene expression, consequently affecting muscle-specific gene expression [93, 94].

Another well-documented effect of *LMNA* mutations causing EDMD is altered PI3-kinase-Akt-Erk2 signaling, which has been demonstrated both in patient cells and in mouse models of the disease (reviewed by Brull et al. [95]). A dramatic effect of this altered signaling has been reported on the cardiac function, and the efficacy of therapies based on inhibition of hyperactivated MAP kinase-Erk1/2 signaling has been experimentally proven [96]. Of note, TGF-*β* 2 and its downstream effectors are increased in EDMD2 mouse models [97] as well as in serum and cells from laminopathic patients eliciting upregulation of fibrogenic molecules [82], a pathogenetic effect relevant to muscle and tendon fibrosis [82].

## 5. Ankrd2 and Lamin in Normal and Laminopathic Muscles

In C2C12 differentiated muscle cells, oxidative stress generated by exposure to H_2_O_2_ induces Akt2 kinase to phosphorylate Ankrd2 at Serine 99 [15]. By using a phosphorylation-defective mutant form of Ankrd2 (Ankrd2 Ser99Ala), we demonstrated that opposite to its wild-type counterpart, phosphorylation-defective Ankrd2 is unable to translocate into the nucleus upon cellular exposure to oxidative stress [98]. Importantly, C2C12 myoblasts expressing Ankrd2 Ser99Ala mutant presented a faster differentiation kinetics [15]. We therefore reasoned that after sensing oxidative stress, Ankrd2 phosphorylation could be required to allow Ankrd2 translocation to the nucleus, where it could participate in the transcriptional modulation of the terminal steps of muscle differentiation. Supporting this hypothesis, we found that Serine 99 is proximal to Ankrd2 NLS (from Lysine 120 to Lysine 123 in the human protein) [15]. Therefore, although the molecular mechanism underlying Ankrd2 nuclear transport is still unclear, upon ROS stimulation, phosphorylation of Serine 99 might unmask the NLS, allowing Ankrd2 to translocate in the nucleus.

**Figure 3:**
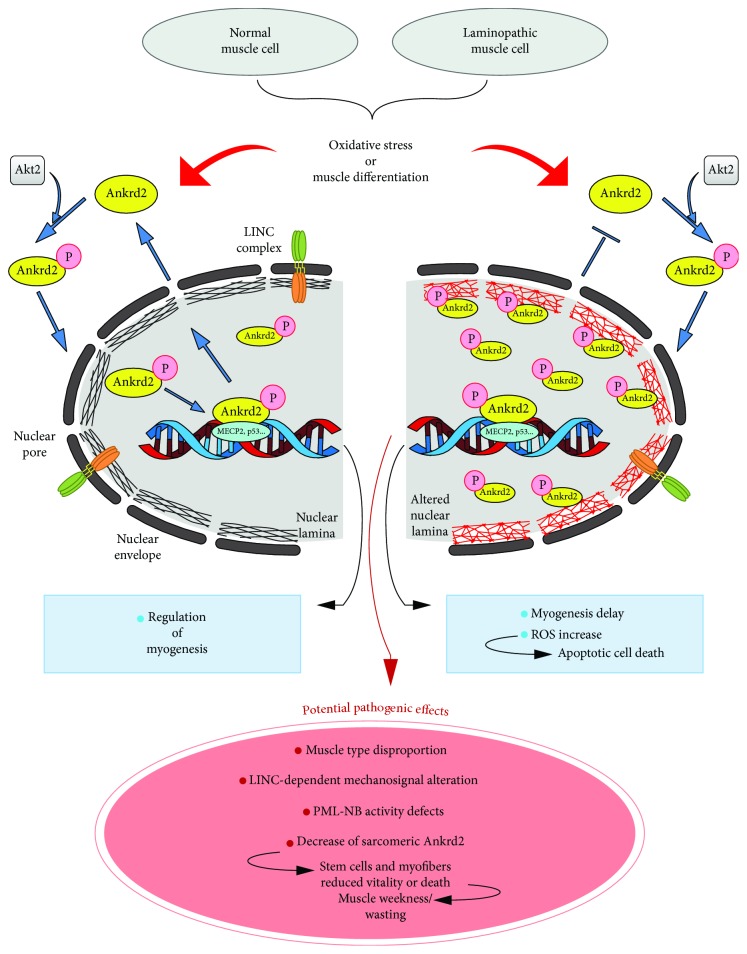
Proposed pathogenic mechanisms in which Ankrd2 might be involved. In healthy muscle cells, physiological amounts of ROS or muscle differentiation induce Ankrd2 phosphorylation by Akt2 [15] and translocation into the nucleus, by binding to A-type lamins (black mesh) [98]. In the nucleus, Ankrd2 modulates the activation of muscle-specific and stress-responsive genes, by interacting with transcription factors (see text and Table 1 for references). In laminopathic muscle cells, mutated A-type lamins (red mesh) even in the absence of external stimuli recruit Ankrd2 into the nucleus. In addition to the already reported consequences, including an anomalous increase of ROS release, myogenic delay, and apoptotic cell death [98], potential pathogenic mechanisms, through which the altered nuclear localization of Ankrd2 might contribute to the pathogenesis of *LMNA*-related muscular disorders, are proposed.

We have demonstrated that during ROS-induced nuclear relocation, Ankrd2 was complexed to A-type lamins, albeit with different affinities for diverse lamin A isoforms [98]. During muscle differentiation, prelamin A levels are increased [61–63], and Ankrd2 interacts with prelamin A with a higher affinity than that established with mature lamin A [98]. Upon the exposure to ROS, Ankrd2 transiently interacted with lamin A and prelamin A, entered the nucleus, and soon after went back to the cytosol. However, EDMD2-linked lamin A mutants constitutively bound Ankrd2, even in the absence of any stimulus, causing Ankrd2 to be retained in the nucleus [98]. We therefore suggest that while under physiological conditions, including muscle differentiation or oxidative stress response, Ankrd2 is transiently retained in the nucleus also due to a reversible increase of prelamin A levels [61, 62, 64, 99], EDMD2-linked lamin A mutants entrap Ankrd2 into the nucleus, affecting its dynamics [98]. Further experiments performed using cells overexpressing Ankrd2 and EDMD2-lamin A mutants revealed that permanent location of Ankrd2 in the nucleus increased release of cellular ROS, as well as apoptotic cell death [98]. Similarly, in laminopathic muscle, the effects of permanent Ankrd2 recruitment in the nucleus might be involved in the reduction of the vitality of myofibers, as well as satellite cells, affecting stem cell renewal and muscle regeneration and resulting in poor adaptation to stress stimuli. A model for a proposed Ankrd2 involvement in the pathogenesis of laminopathies is depicted in Figure 3.

Although direct observations of Ankrd2 localization and functions in EDMD2 muscle (including nascent, mature, or regenerating fibers) are still missing, we can suppose that the catastrophic effects of mutated A-type lamins in muscular laminopathies might be also due to the altered Ankrd2-lamin A interaction affecting both mechanosensing and transcriptional activity of Ankrd2.

## 6. Perspectives for the Study of Lamin A-Ankrd2 Interplay

### 6.1. Mechanosignaling Regulation through Ankrd2

Albeit still unexplored, a consequence of an altered nuclear recruitment of Ankrd2 observed in laminopathies might also be related to a reduced availability of cytoplasmic Ankrd2 at the I-bands of the sarcomere and thus to a decreased mechanosensing activity. Therefore, it would be worth evaluating the change in the Ankrd2 amount at the sarcomere in laminopathic conditions. Moreover, given the involvement of lamin A in mechanosignaling through the LINC complex, it would be worth deepening the potential involvement of Ankrd2 in lamin A/LINC complex-dependent mechanisms of response to mechanical strain.

### 6.2. Muscle Fiber Type Disproportion (FTD)

Muscle fiber type disproportion (FTD) is a pathological condition in which there is the selective reduction of the size of slow and not fast myofibers. FTD is observed in many cardiac and skeletal muscle disorders, with severe effects on the functionality of all the striated muscle. Interestingly, since Ankrd2 is principally expressed in slow fibers, pathological nuclear recruitment of Ankrd2 might also be involved in the damage and atrophy of slow fibers reported in muscular laminopathies [100, 101].

In fact, FTD has been observed in some cases of LGMD1B [100, 102] as well as EDMD2 [100]. Furthermore, in skeletal muscle from Lmna^H222P/H222P^ mice, in addition to FTD, atrophy of fast fibers was detected [101].

### 6.3. PML-NB

Promyelocytic leukemia protein-nuclear bodies (PML-NBs) are nuclear matrix-associated domains recruiting a variety of seemingly unrelated proteins with the aim of regulating many cellular functions from transcriptional regulation to growth regulation, tumor suppression, apoptosis, and storage of nuclear proteins under stress conditions [103]. PML-NBs are presumed to mediate protein-protein interactions and function as a platform that promotes protein posttranslational modification, for example, SUMOylation, acetylation, ubiquitination, and phosphorylation [104]. This has been demonstrated for p53, which is activated in PML-NBs of C2C12 myoblasts, upon exposure to ROS [105]. Interestingly, both Ankrd2 and lamin A/C bind to PML, a core constituent of PML-NBs, and albeit not essential for PML-NB function, it regulates their development under stress condition [106]. Since it has been reported that progerin, the truncated and toxic prelamin A responsible for the progeroid disease HGPS, progressively accumulated in PML bodies, altering their shape and number [107], it is therefore possible that other lamin A/C mutants might participate in the formation of PML-NBs under stress conditions, perturbing their downstream pathways involved in transcription, apoptosis, senescence, response to DNA damage, etc. As already reported, Ankrd2 localizes at PML-NBs under stress conditions [1]. The possibility that mutant A-type lamins and Ankrd2 localization at the PML-NBs concur to aberrantly regulate PML-NB activity under stress conditions warrants further investigation.

### 6.4. Therapeutic Perspectives

Sequestration of proteins at the nuclear envelope or inside the nucleus by lamin mutants as well as by excess levels of wild-type lamins has been widely reported and involves DNA-binding proteins such as BAF and transcription factors such as Oct-1, c-FOS, and AP-1 [108–118]. An interesting condition has been described in a *Drosophila* model of EDMD2 as well as in patient muscle fibers, where nuclear accumulation of the stress-responsive protein Nrf2 (Nuclear factor erythroid 2-related factor 2) was observed [119]. It is conceivable that Nrf2 and Ankrd2 aberrant dynamics both contribute to altered stress response in EDMD2. The identification of molecules able to release aberrant interactions between mutated lamins and their binding partners, including Ankrd2, may provide innovative therapeutic tools for laminopathies.

## 7. Concluding Remarks

In conclusion, although direct evidence on Ankrd2 localization in forming, mature, and regenerating EDMD2 muscle fibers is still missing, we hypothesize that alterations in expression, PTMs, interactors, or nuclear localization of Ankrd2 might contribute to impair the response to mechanical and/or oxidative stress. Future studies exploiting different laminopathic animal models, including murine Lmna^H222P/H222P^ KI, Lmna^∆K32^ KI, or Lmna KO (respectively, reproducing conditions very close to human cardiomyopathy, L-CMD and EDMD [101, 120, 121]), would enable further novel insights into the role of Ankrd2 in the pathophysiology of muscular laminopathies.

Finally, a proteomic analysis of Ankrd2 interactome in mature muscle (healthy and EDMD2-affected), as well as the creation of animal models expressing unphosphorylatable Ankrd2 forms, will help to better define the role of Ankrd2 in muscular tissue, allowing the assignment of a specific role of oxidative stress-dependent phosphorylation to the pathology of other cardiac and skeletal muscle disorders.

## Figures and Tables

**Table 1:** Overview of the main structural and functional features of Ankrd2 (extensively reported in [122]).

Feature	Ref.
*Structural domains*	
Ankyrin repeats	[123]
Coiled coil	
Protein destabilization motif (PEST)	
Nuclear localization signal (NLS)	
*Posttranslational modifications*	
Phosphorylation by Akt2 kinase	[15]
Proteolytic cleavage by *μ*-calpain	[9]
*Expression profile*	
Skeletal muscle > heart > kidney	[4, 19, 123, 124]
Type I (slow) skeletal muscle fibers	[8, 123]
Localized in nuclei of myoblasts and cytoplasm of myotubes	[123]
Migrate from myofibrils to nucleus after muscle injury	[10]
*Proposed functions*	
Stress response in muscle (stretch, denervation, eccentric contractions, fatiguing jumping and other types of exercise, and injury)	[8, 10, 16, 46, 47, 49, 50, 55]
Muscle adaptation	[7, 10, 16, 20, 46, 47, 49, 50, 125, 126]
Transcriptional regulation	[1, 10]
Communication between the sarcoplasm and the nucleus	[1]
Myogenesis and myogenic differentiation	[11, 13, 14]
Inflammatory response	[12]
*Interacting proteins*	
Titin	[7]
Telethonin	[1]
ZASP	[30]
Calpain 3	[9]
Akt2	[15]
A-type lamins (lamin A, prelamin A, and lamin C)	[98]
Proteins with PDZ and SH3 domains, including ZO-1 and *β*1-syntrophin	[20]
Transcription factors, including YB1, p53, PML, PAX6, NFIL3, and MECP2	[20]
*Pathways affected by Ankrd2 silencing in human myoblasts*	
Intercellular communication	[20]
Cytokine-cytokine receptor interaction	
Focal adhesion	
Tight junction	
Gap junction	
Regulation of actin cytoskeleton	
Signaling involved in intracellular communication	
Calcium	
Insulin	
MAPK	
p53	
TGF-*β*	
Wnt	
